# Pigtailed macaques as a model to study long-term safety of lentivirus vector-mediated gene therapy for hemoglobinopathies

**DOI:** 10.1038/mtm.2014.55

**Published:** 2014-12-17

**Authors:** Hans-Peter Kiem, Paritha I Arumugam, Christopher R Burtner, Catherine F Fox, Brian C Beard, Phillip Dexheimer, Jennifer E Adair, Punam Malik

**Affiliations:** 1 Fred Hutchinson Cancer Research Center, Seattle, Washington, USA; 2 University of Washington Medical School, Seattle, Washington, USA; 3 Division of Experimental Hematology and Cancer Biology, Cancer and Blood Diseases Institute, Cincinnati Children’s Hospital Medical Center, Cincinnati, Ohio, USA; 4 Divisions of Human Genetics and Bioinformatics, Cincinnati Children’s Hospital Medical Center, Cincinnati, Ohio, USA

## Abstract

Safely achieving long-term engraftment of genetically modified hematopoietic stem cells (HSCs) that maintain therapeutic transgene expression is the benchmark for successful application of gene therapy for hemoglobinopathies. We used the pigtailed macaque HSC transplantation model to ascertain the long-term safety and stability of a γ-globin lentivirus vector. We observed stable gene-modified cells and fetal hemoglobin expression for 3 years. Retrovirus integration site (RIS) analysis spanning 6 months to 3.1 years revealed vastly disparate integration profiles, and dynamic fluctuation of hematopoietic contribution from different gene-modified HSC clones without evidence for clonal dominance. There were no perturbations of the global gene-expression profile or expression of genes within a 300 kb region of RIS, including genes surrounding the most abundantly marked clones. Overall, a 3-year long follow-up revealed no evidence of genotoxicity of the γ-globin lentivirus vector with multilineage polyclonal hematopoiesis, and HSC clonal fluctuations that were not associated with transcriptome dysregulation.

## Introduction

Genotoxic adverse events have been observed in clinical trials using γ-retroviral vectors (RV) for genetic modification of hematopoietic stem cells (HSC).^1–5^ The clinical experience with lentivirus vectors (LV), although in its infancy, has not been met with the same complications.^6–8^ Although murine studies and the *in vitro* immortalization assay indicate an overall safer profile of LV compared to RV,^9–12^ emergence of clonal dominance from a gene-modified HSC due to dysregulation of the *HMGA2* gene in a β-thalassemia patient treated with a β-globin LV was observed.^13^ The hematopoietic contribution from this clone was stable for 2–3 years, and is now reportedly declining.^14^ Since clonal dominance in past RV trials, although initially therapeutic,^15^ evolved to monoclonal expansion and malignancy,^2^ this caused significant concern and warrants more clinically applicable modeling of human hematopoiesis.

Nonhuman primates have similarities to humans in their gene structure/expression profile,^16^ HSC engraftment/cycling kinetics, and allow a robust analysis of proviral integration sites in large numbers of HSC.^17–22^ In rhesus macaques, the innate host restriction factor tripartite motif-containing protein 5α (TRIM5α) prevents retroviral infection by degrading the viral capsids.^23,24^ In human and rhesus HSCs, TRIM5α variations strongly influenced LV transduction efficiency.^25^

We have previously described efficient transduction of pigtailed macaque long-term repopulating cells using LV;^26^ and also reported that the self-inactivating (SIN) γ-globin LV, sGbG, ameliorates the sickle cell disease in mice.^27^ Using the *in vitro* immortalization assay, this LV transcriptional unit showed ~200-fold lower genotoxic potential in primary murine hematopoietic stem/progenitor cells compared to LV carrying strong viral promoter/enhancer elements.^10^ Herein, we report the long-term follow-up of a macaque transplanted with CD34^+^ HSC genetically modified with this clinically relevant γ-globin-expressing LV.

## Results

We transplanted a juvenile pigtailed macaque (A09172) with HSCs transduced with sGbG, a SIN lentiviral vector expressing γ-globin gene under the control of erythroid-specific β-globin promoter and strong globin regulatory elements. A second juvenile macaque (A09159) was transplanted at approximately the same time with HSCs transduced with two SIN lentiviral vectors expressing GFP/YFP served as a control. Following myeloablative total body irradiation and hematopoietic stem cell transplant (HSCT), the sGbG macaque (A09172) displayed the anticipated drop in blood counts, which recovered by 3 weeks due to HSC engraftment and was comparable to the control animal (A09159) (Supplementary Figure S1). In the macaque which received autologous sGbG-transduced HSC, 15.8% of the cells were positive for viral integration in liquid culture on day 11 after transduction. In contrast, HSC from the animal serving as control were 4.1% positive for the GFP arm and 3.3% for the YFP arm. Low transduction efficiency (<1% in both arms) in the control macaque likely contributed to low engraftment of gene-modified cells following transplant in this animal. This control macaque, therefore, was only used as a transplant and HbF control and was not further analyzed for long-term gene marking and integration.

**Figure 1:**
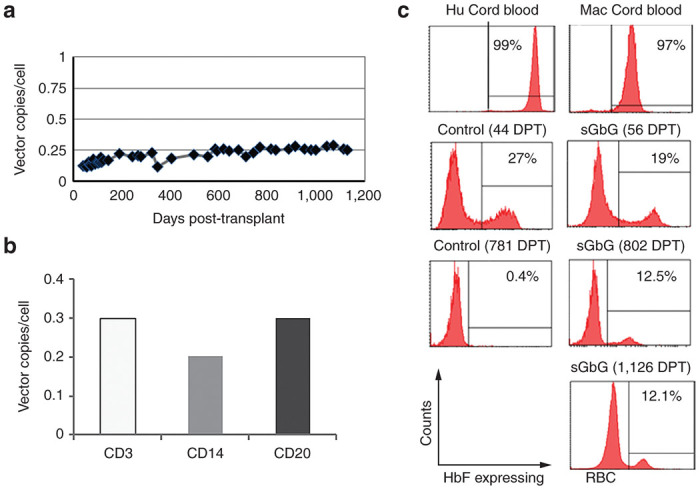
Human γ-globin gene marking and sustained long-term expression of human fetal globin (HbF) expressing red blood cells (RBCs) in γ-globin LV (sGbG) transplanted macaque. (**a**) Vector copies/cell in the circulating peripheral white blood cells (WBCs) of the sGbG macaque at various days post transplantation (DPT). (**b**) Vector copy number analysis on different hematopoietic lineages CD3 (T cell lymphocytes), CD14 (a marker nonhuman primate granulocytes), and CD20 (B cell lymphocytes). (**c**) Representative histogram plots showing HbF-expressing RBCs from human (Hu) and macaque (Mac) cord blood controls, a transplanted control macaque and sGbG macaque at early (44 DPT and 56 DPT) and late (781 DPT, 802 DPT and 1,126 DPT) time-points, respectively. The human HbF antibody detects human γ-globin complex, also crossreacts with macaque HbF (macaque α_2_: human γ_2_ globin complex, as in RBCs expressing sGbG, or with macacque α_2_: macaque γ_2_ complex or macaque HbF). At every time-point analyzed, human umbilical cord blood positive controls, and early and late transplant macaque controls were included in HbF staining.

Longitudinal analysis for vector copy number (VCN) in peripheral blood leukocytes from the sGbG macaque showed stable gene marking for over 3.1 years in peripheral blood leukocytes at ~25% (Figure 1a). In addition, 7.5% of bone marrow colony-forming cells were found to be gene modified at 2 years posttransplantation. Additionally, gene transfer was present at the same level in multiple hematopoietic lineages: VCN analysis on different lineages CD3 (T cell lymphocytes), CD14 (marker non-human primate granulocytes), and CD20 (B cell lymphocytes) showed vector copies of 0.3, 0.20, and 0.3, respectively (Figure 1b). The purity of the sorted cell lineages CD3, CD14, and CD20 were found to be >94% by flow cytometry (Supplementary Figure S2). An upregulation of fetal hemoglobin was observed in the red blood cells of the control and sGbG macaque early after transplantation. But, the HbF expressing red blood cells in control macaque declined to 0.4%, while the levels of HbF were maintained at 12.5–13% in the sGbG macaque for over 3 years (Figure 1c).

**Figure 2:**
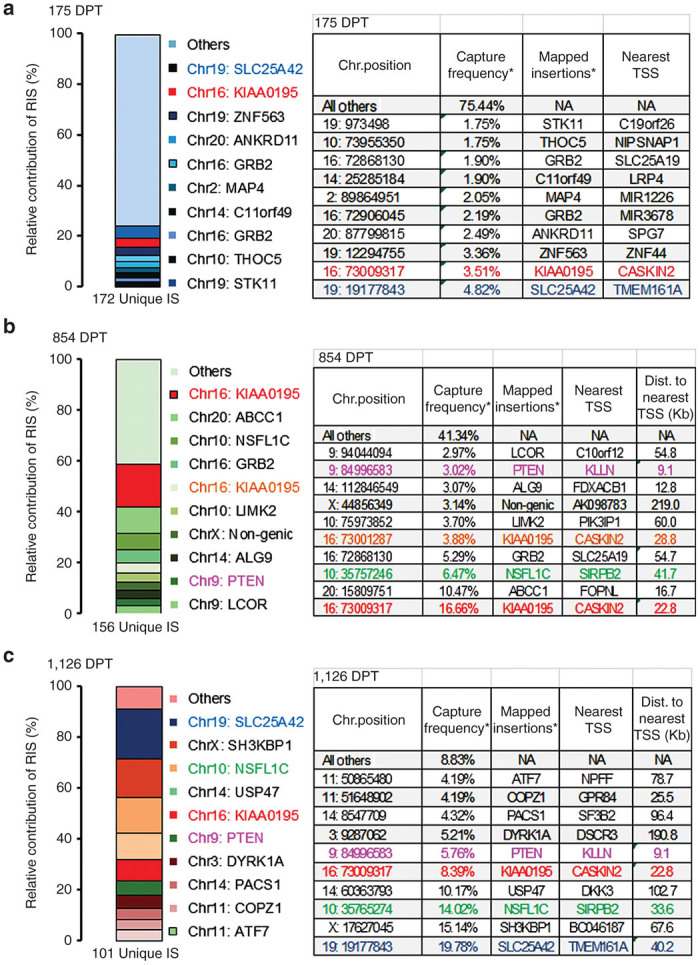
Longitudinal integration site analysis in the sGbG vector transplanted macaque. (**a**) The top 10 most abundantly retrieved insertions are denoted in distinct colors, each color representing a top 10 retrieved RIS at 175 days post-transplant (DPT), (**b**) 854 DPT, and (**c**) 1,126 DPT. “All others” at the top of the column represent vector insertions that are less frequent. The only insertion that was retrieved at all three time-points analyzed is indicated in red, and mapped within the intron of *KIAA0195* gene (Chr16: 73009317). The surrounding gene *CASKIN2* is located within ~30 kb of this insertion site. A second integration in *KIAA0195*, ~8 Kb from the first, was discovered at 854 DPT, and is indicated in orange. Among the top 10 insertions, SLC25A42, PTEN, and NSFL1C were retrieved at more than one time point. Details of top 10 retrieved insertions and surrounding genes at three time-points (175, 854, and 1,126 days post-transplant) in the sGbG vector-transduced macaque are shown.

Long-term follow-up allowed for a thorough evaluation of the retroviral integration site (RIS) profile in gene-modified hematopoietic cells up to 3.1 years after transplant. High-throughput RIS analysis in blood revealed a total of 376 unique RIS present in peripheral blood cells between 6 months and 3.1 years after transplant (Figure 2). The proviral insertions were distributed over all chromosomes, with a slight overrepresentation into chromosome 14 and chromosome 19 at later time point (Supplementary Figure S3a). We did not observe clustering of sGbG LV vector insertions near transcriptional start sites (Supplementary Figure S3b–d); 63% of integrations were found within transcriptional units. To assess clonality, we examined the top 10 most frequently captured RIS at each time point, which were detected at frequencies ranging from 1.75 to 19.78% (Figure 2a–c).^28^ Among the retrieved RIS, only 2.7% (10/376) of the clones were present at all three time points (Supplementary Table S1) at different retrieval frequencies; and 11.4% (43/376) of the clones were present at two time points. Notably, for each of the three time points analyzed, 83, 75.6, and 73.3% of the LV insertions were unique.

Notably, one RIS that was retrieved at all three time points represented 3.5% of detected RIS at 175 days posttransplant (DPT), 16.7% at 854 DPT, and 8.4% at 1,126 DPT (Figure 2a–c). This RIS mapped within the intron of an uncharacterized gene, *KIAA0195*, ~22.8 kb away from the nearest transcription start site (TSS) of *CASKIN2*. A second integration which constituted one of the top 10 most frequently captured clones at 175 and 1,126 DPT mapped to *SLC25A42*, a nuclear-encoded mitochondrial protein, which lies 40.2 kb away from transmembrane protein 161A (*TMEM161A*). This integration was also sequenced at 854 DPT, but at a frequency less than 0.01% of all integrations.

Two genes (*PTEN* and *PACS1*) that were sequenced at low frequencies 175 and 854 DPT were among the top 10 retrieved insertions at 1,126 DPT and were present at frequencies of 5.8 and 4.3%, respectively (Supplementary Table S1 and Figure 2a–c). However, multiple vector insertions were absent in these gene loci. Additionally, a single integration in the *NSFL1C* gene on chromosome 10 increased from 6.47% at 854 DPT to 14.02% at 1,126 DPT but was not sequenced at the first time point.

**Figure 3:**
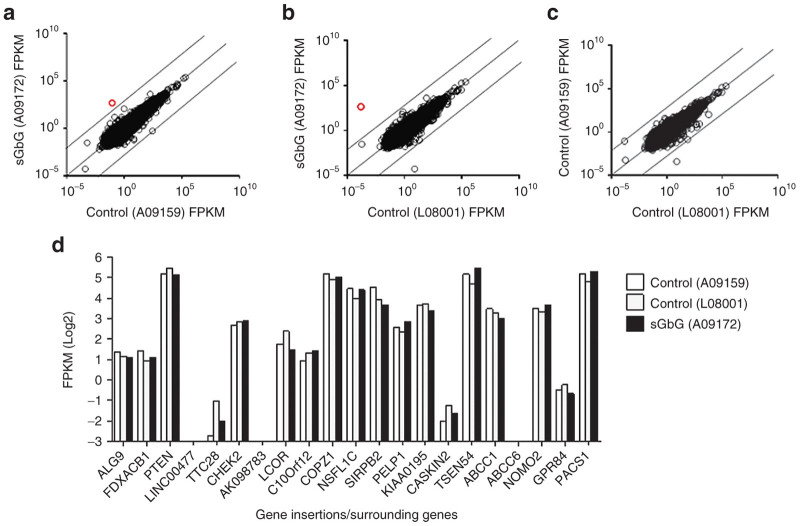
Gene expression profile in control macaques and the macaque transplanted with HSC transduced with sGbG vector. (**a–d**). Comparison of transcriptome profiles determined by mRNAseq, expressed as fragments per Kilobase of exon per Million fragments mapped (FPKM) between (**a**) sGbG and A09159 lentiviral control macaque, and (**b**) sGbG and a L08001 nontransplanted control macaque, and (**c**) the two control macaques. Transcript levels of >30,000 genes were analyzed. FPKM values are represented in empty black circles. Empty red circles in Figure 2a and 2b represent higher expression of microRNA-650a-2 (FPKM:465) in the sGbG macaque compared to the two control macaques. (**d**) Gene expression levels of the top 10 gene insertions/surrounding sites retrieved in the sGbG macaque compared to the two control macaques. The values are plotted in log2 scale.

To assess the transcriptional consequences of sGbG integrations *in vivo*, we performed whole transcriptome sequencing (mRNAseq) to get the absolute and relative levels of transcripts in whole blood at 935 DPT. mRNAseq from the transplanted control macaque (A09159) and a third control macaque that did not receive HSC transplant (L08001) was performed concurrently. No differences in global gene expression profile were detected between the sGbG macaque and the LV-control and nontransplanted macaques (Figure 3a–c), similar to what has been reported for rhesus macaques receiving SIV-based LV.^29^ The transcript levels of all genes flanking RIS were not significantly different from those of two control macaques. Specifically, the transcript levels of *CASKIN2*, which mapped close to the frequently captured RIS within *KIAA0195*, differed from the control macaques by less than twofold. The expression of genes PTEN (tumor-suppressor), COPZ1 (involved in autophagy and protein trafficking), and GPR84 (G-protein coupled receptor) were not significantly altered due to vector integrations (Figure 3d). We observed an upregulation of miR650a-2 in the transcriptome of the sGbG macaque, compared to control macaques.

## Discussion

In this study, long-term follow-up of a pigtailed macaque transplanted with a lentiviral vector carrying a human γ-globin gene showed stable gene transfer, polyclonal hematopoietic reconstitution, and sustained human fetal globin expression 3 years following bone marrow transplantation. Notable were hematopoietic clonal fluctuations of lentivirally marked HSC which led to temporary clonal dominance in the absence of gene dysregulation or emergence of oligo- or mono-clonality or hematopoietic malignancy. Our data demonstrates the safety of genetic manipulation with globin carrying lentivirus vectors in a large animal model.

Strategies for human gene therapy trials targeting HSC in hemoglobinopathies are limited by studies in murine models, which have differences in stem cell kinetics, and current xenograft models do not support human erythropoiesis. Nonhuman primates closely predict results of human HSC transplantation in terms of the number of engrafted gene-modified cells that are required for stable polyclonal hematopoiesis.^30,31^ Macaques also serve as reliable models for studying fetal globin regulation in humans. Currently, there is no large animal model available for sickle cell disease or thalassemia. The aforementioned factors are critical in modeling gene therapy for hematological diseases like sickle cell anemia and β-thalassemia, where the level of globin gene expression required for therapeutic correction is comparatively higher compared to other inherited disorders and hence genetic modification is needed in a therapeutic number of engrafted HSC.

As seen in human patients following a HSC transplant,^24,25^ we observed an upregulation of fetal globin (HbF) in red blood cells (RBC) of the control macaque and the sGbG macaque early after transplantation.^32,33^ Importantly, while the number of HbF expressing RBCs in the control macaque declined to 0.4% by 2 years, the HbF expressing RBCs were maintained at 12.5–13% in the sGbG macaque for over 3 years. This level of HbF expression was achieved despite presumptive competition between the endogenous macaque β-globin chains and the LV-derived human γ-globin chains for macaque α-globin, which would not occur in the case of thalassemia major. Stable vector copy number over the 3-year period and comparable vector copies in different hematopoietic lineages strongly support long-term stable engraftment of gene-modified long-term repopulating HSCs, which are capable of multilineage repopulation.

The pattern of integration sites in the sGbG macaque was consistent with that reported for other lentiviral gene therapy vectors.^34^ Sixty-three percent of insertions were in/around genes, and integrations did not cluster near transcription start sites (TSS), contrasting with RV that frequently insert within 2.5 kb of TSS.^35^ We did not retrieve more than two independent vector insertions in the KIAA0195 genomic locus within a 50 kb window. No insertions were found in/near the predicted *LMO2*, *MDS/EVI1*, *PRDM16*, or *HMGA2* gene sequences; insertional oncogenesis has been associated with the former three genes with γ-retroviral vectors in several trials, and insertional gene dysregulation of *HMGA2* has been seen to occur with a β-globin expressing lentivirus vector due to aberrant splicing from the insulator within the vector to a natural splice acceptor in the *HMGA2* intron.^2,15,36–38^ These data strongly support a dynamic clonal fluctuation of hematopoietic contribution from gene-modified HSCs, without evidence of clonal dominance. Our results are consistent with reports from clinical trials with LV for X-linked adrenoleukodystrophy, Wiskott–Aldrich syndrome, and metachromatic leukodystrophy, which revealed polyclonal reconstitution of engrafted cells from a dynamic clonal output, without any evidence of *in vivo* clonal expansion of gene-corrected cells, suggesting safer gene transfer strategy with SIN-LV.

MicroRNA 650 lies in the immunoglobulin gene locus, and its upregulation has been found to be associated with better prognosis in chronic B-cell leukemia.^39^ No RIS in/around the gene encoding this microRNA was found using the current method. The role of LV in dysregulation of miR 650-a2 or the significance of this upregulation is therefore currently unknown.

Taken together, the expression of genes in and around all the γ-globin LV insertions was largely unperturbed. The stable copy number in all lineages, sustained HbF expression in red blood cells, and hematopoietic contribution from different clones at multiple time points without alterations in transcriptome suggest HSC clonal fluctuations. Furthermore, our analyses do not reflect clonal expansion or insertional mutagenesis. Similar RV marked HSC clonal fluctuations were shown in mice^11^ and in a human receiving cells genetically modified with an anti-HIV LV.^40^

In conclusion, long-term follow-up of a macaque transplanted with hematopoietic stem cells gene-modified with a human γ-globin-expressing LV demonstrates a relatively safe profile and potential therapeutic utility of this vector. These results are based on the characterization of stable hematopoietic reconstitution, polyclonal gene-modified hematopoiesis, and stable transgene expression. Absence of insertions leading to pronounced clonal outgrowth or transcriptional dysregulation has promising implications for clinical LV-based gene therapy.

## Materials and Methods

### Gene transfer and transplant

Pigtailed macaques are housed at the Washington National Primate Research Center using appropriate regulatory approvals. The macaques reported in this study (A09172 and A09159) were primed with G-CSF (100 μg/kg) and SCF (50 μg/kg) daily for 5 days, prior to bone marrow aspirate. After the bone marrow cell harvest, each macaque received a fractionated dose of 1020cGy total body irradiation on day -2 and day -1 of transplant. Bone marrow was lysed in hemolytic buffer and CD34^+^ cells were isolated by incubating with CD34 antibody (clone 12.8), produced in-house, and subsequently separated by immune-magnetic techniques with anti-mouse IgM microbeads (Miltenyi Biotec, San Diego, CA). Cells from A09172 were transduced twice with the SIN sGbG lentiviral vector (LV) at a MOI 10 and 8, using methods previously described.^26,27^ Cells from A09159 were divided into two arms, and similarly transduced twice with (i) a SIN LV expressing GFP at an MOI of 10 or (ii) a SIN LV expressing YFP at a MOI of 9.65. Following the transduction procedure, the cells were washed and infused back into the animal at 29 × 10^6^ cells/kg for sGbG macaque (128 × 10^6^ total cells) and 89 × 10^6^ cells/kg for the control macaque (320 × 10^6^ total cells). Standard supportive care post-transplant was provided as needed.^26^

### Vector copies and expression

Genomic DNA from peripheral blood leukocytes and different hematopoietic lineages were subjected to Taqman quantitative polymerase chain reaction analysis with primers/probes spanning the R and U5 region of the lentiviral long-terminal repeat (Supplementary Table S2) on the Applied Biosystems 7900HT. HbF staining is described in Supplementary Methods.

### RIS analysis

Modified genome sequencing polymerase chain reaction was performed for RIS analysis as described,^41^ using primer-probes listed in Supplementary Table S2. Genomic mapping of RIS was carried out by aligning flanking genomic sequences to the rhesus genome (rheMac3) to determine the locus. To assign nearest genes, rhesus alignments were converted to the corresponding loci within the human genome (hg19) using the UCSC genome browser conversion tool (www.genome.ucsc.edu/).

### Transcriptome analysis

RNA was extracted from blood using TRIzol (Invitrogen Life technologies, Carlsbad, CA) and mRNAseq was performed at Cincinnati Children’s Genomics Core Facility and analyzed as described.^42–44^
