# Supplementary figures and images for: Pigtailed macaques as a model to study long-term safety of lentivirus vector-mediated gene therapy for hemoglobinopathies
Source: Mol Ther Methods Clin Dev. 2014 Dec 17;1:14055–. doi: 10.1038/mtm.2014.55 (PMC4448740; doi:10.1038/mtm.2014.55)

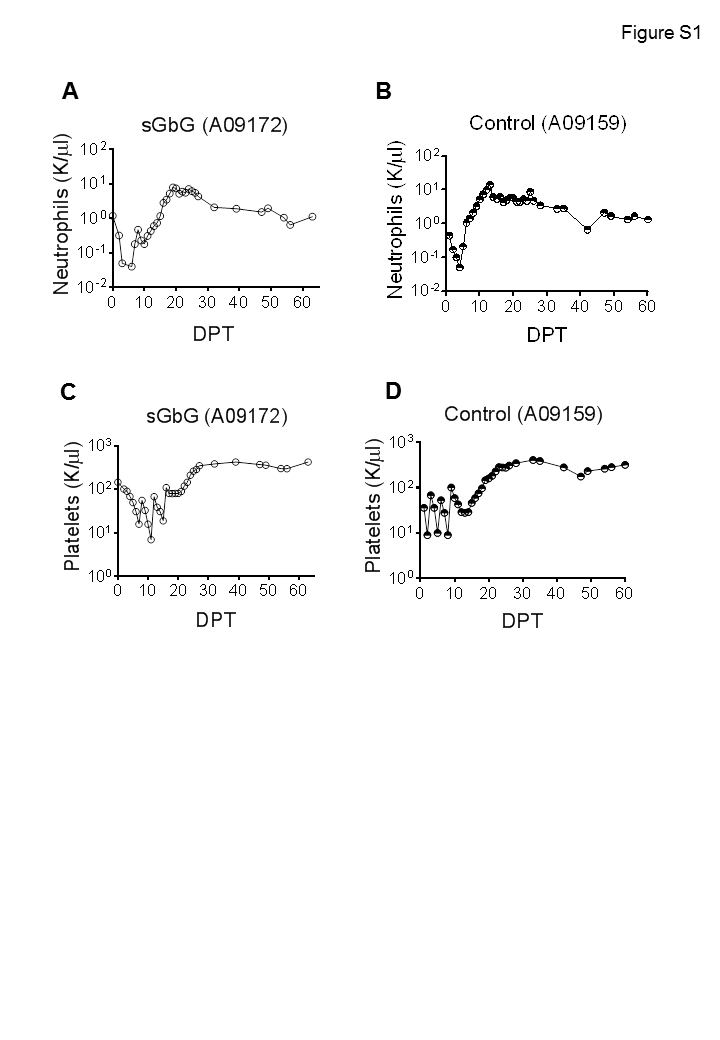

Supplement: Supplementary Figure S1 [file mtm201455-s1.tiff]

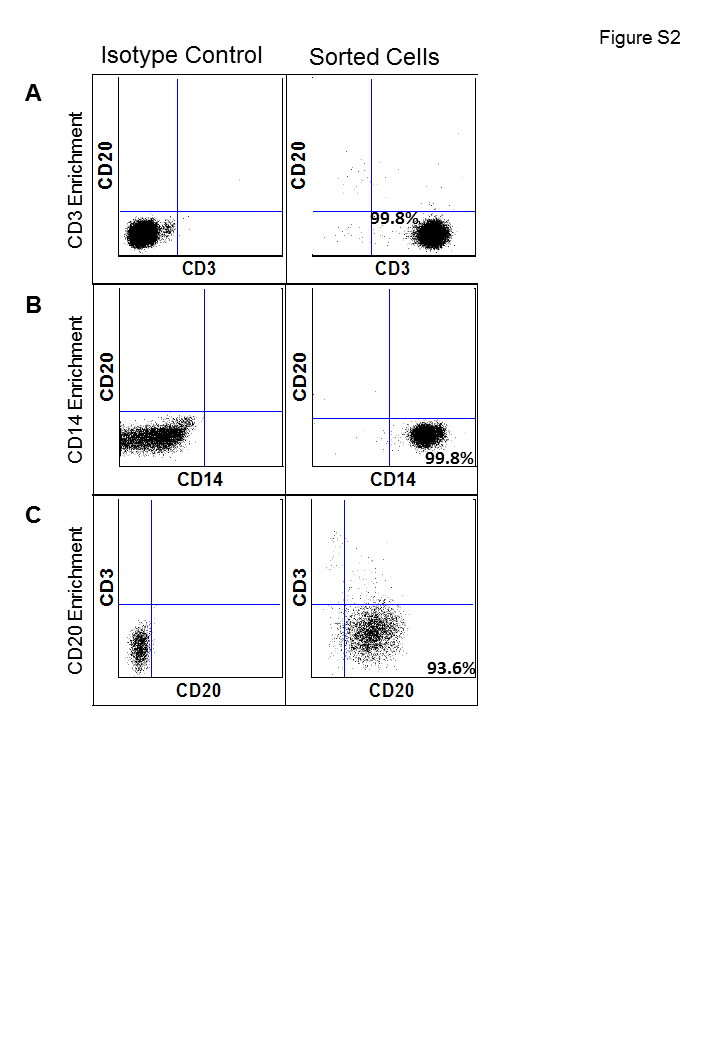

Supplement: Supplementary Figure S2 [file mtm201455-s2.tiff]

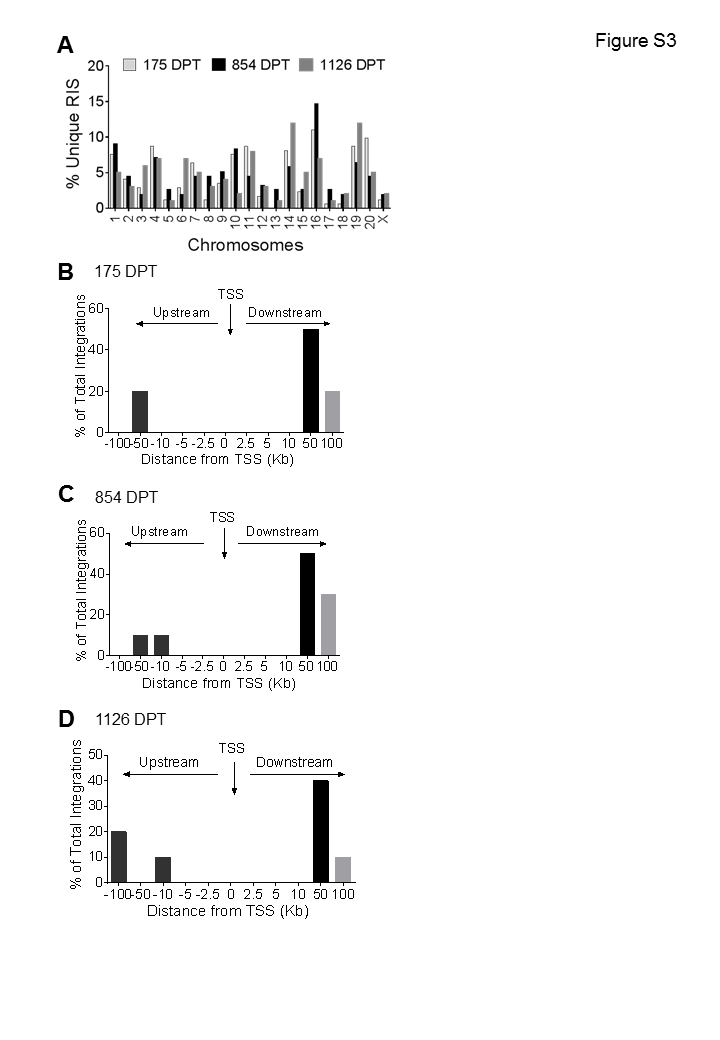

Supplement: Supplementary Figure S3 [file mtm201455-s3.tiff]
