# Supplementary material for: Pigtailed macaques as a model to study long-term safety of lentivirus vector-mediated gene therapy for hemoglobinopathies
Source: Mol Ther Methods Clin Dev. 2014 Dec 17;1:14055–. doi: 10.1038/mtm.2014.55 (PMC4448740; doi:10.1038/mtm.2014.55)
Supplement: Supplementary Tables [file mtm201455-s4.doc]

**Supplementary Table**

**
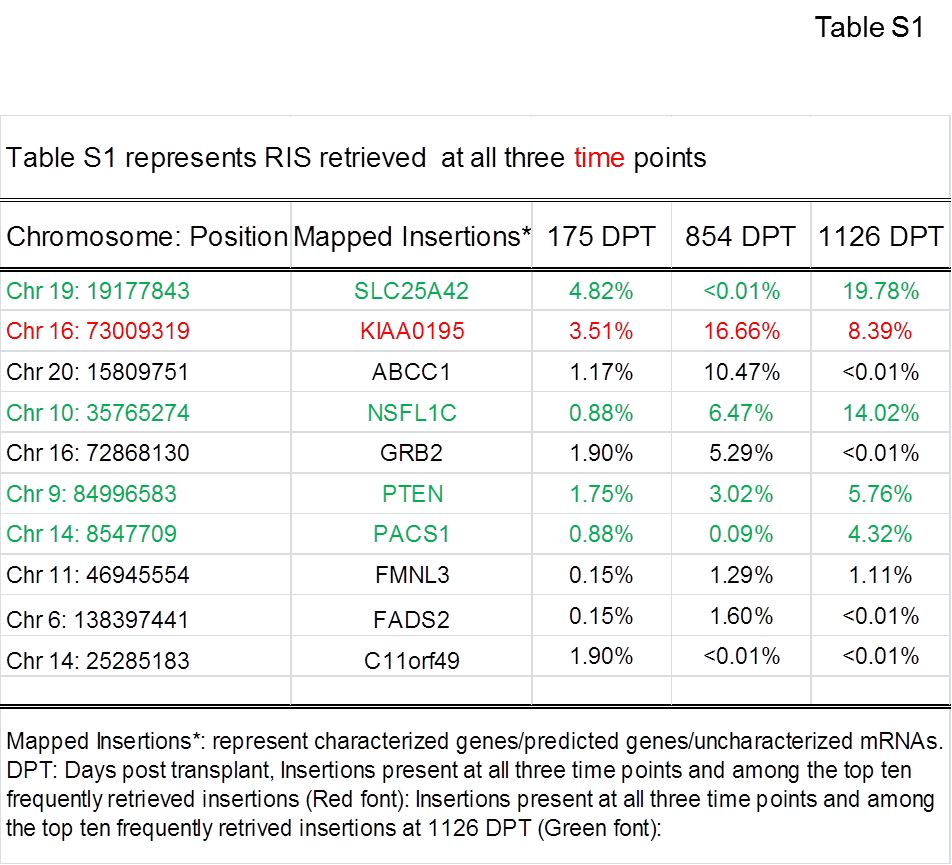
**

**Table S2**. Real-time PCR primers for copy number analysis and primers

used for genome sequencing PCR
