# Supplementary material for: Pigtailed macaques as a model to study long-term safety of lentivirus vector-mediated gene therapy for hemoglobinopathies
Source: Mol Ther Methods Clin Dev. 2014 Dec 17;1:14055–. doi: 10.1038/mtm.2014.55 (PMC4448740; doi:10.1038/mtm.2014.55)
Supplement: Supplementary Methods [file mtm201455-s5.pdf]

Supplementary Materials and Methods:

sGbG LV production. sGbG lentivirus vector encodes the human γ-globin gene and its construction has been previously described.(1) sGbG virus was produced by transient cotransfection of 293T cells using the sGbG plasmid, packaging Δ8.9, and envelope plasmids (VSV-G). The virus-containing medium was harvested at 24 hours and 48 hours post transfection, concentrated by ultracentrifugation (25,000 rpm × 90 minutes) using a Beckman Coulter Optima L-90K Ultracentrifuge (Brea, California). Viral titers were determined by infecting mouse erythroleukemia (MEL) cells with serially diluted lentivirus, differentiating them, and analyzing HbF expression by fluorescence-activated cell-sorter scanner (FACS), as described.(1)

HbF staining: LV transduced MEL cells were fixed in 60μL 4% paraformaldehyde for an hour at room temperature, washed with phosphate-buffered saline (PBS). Fixed cells were blocked using 75μL of 5% nonfat milk for 10 minutes at room temperature. After washing in PBS, the cell pellet was permeabilized with 10 μL Caltag Reagent B (Caltag Laboratories, Burlingame, CA). A three step staining protocol was applied. First, purified mouse anti-human fetal hemoglobin (1μg/10^6 cells) antibody (BD Biosciences, San Jose, California) was added, followed by Biotin rat anti-mouse IgK light chain antibody (BD Biosciences) and the HbF was detected using Streptavidin PE (eBiosciences, Santa Clara, California). Cells were incubated for 30 minutes at room temperature in dark, after addition of each antibody and washed in PBS. Stained cells were analyzed using FACS Canto II (BD Biosciences). **RBC** (2.5 x10^7 cells) were fixed with 0.05% glutaraldehyde for 10 minutes (min) at room temperature, washed thrice with PBS and resuspended in 0.1% TritonX-100 for 3-5 min. The cells were washed and approximately 2x10^5 cells were stained with 5μL of HbF monoclonal antibody for 15 min at room temperature, washed and analyzed by flow cytometry. Cord blood samples were used as a positive control and age-matched appropriate transplant controls were stained simultaneously.

Modified genome sequencing PCR

Sheared genomic DNA (sheared to ~1500 bp) product was polished with DNA End Repair Mix (Life Technologies, Grand Island, NY) and modified linkers were ligated following the manufacturer's directions (454/Roche-GS 20 DNA library preparation kit); 100–200ng of DNA was amplified in sequential nested exponential PCRs using primers listed in Table 1:

Specific products from the first nested PCR were captured and washed by the biotin tag, and DNA was diluted 1:100 in H_2O before the second nested PCR. DNA fragments in the range of 300-800bp were gel-purified and sequenced by Ion Torrent® semiconductor sequencing available from Edge Biosciences (Gaithersburg, MD) following standard procedures.

Genome mapping of insertion sites

The resulting junction sequences were aligned to version 2 (rheMac2) of the rhesus genome with a stand-alone version of BLAT that generates a BLAST alignment score. To assign the closest transcription start site (TSS) proximal to the site of virus integration, the flanking genomic sequence was converted to the corresponding location within the human genome (assembly GRCh37/hg19) using the UCSC genome browser genome conversion view.

mRNAseq Processing

1µg of total RNA was polyA selected and reverse transcribed using Illumina's TruSeq RNA library preparation kit V2. Each sample can be fitted with one of 24 adapters containing a different 6 base molecular barcode for high level multiplexing. After 12 cycles of PCR amplification, completed libraries are sequenced on an Illumina HiSeq2000, generating around 20 million high quality 50 base long reads per sample. RNA-Seq analysis and processing of reads were done as described previously. RNA sequence analysis is based on the TopHat (2.0.8b)/Cufflinks (V2.1.1) pipeline. First, sequences were aligned to the reference genome with TopHat,[14] which efficiently aligns reads spanning known or novel splice junctions. Each sample was then independently processed with Cufflinks[15] in order to generate an initial transcriptome. Cuffmerge tool was used to merge the private transcriptomes into a single reference, and at the same time annotate known genes and extend partial transcripts.[16] This common transcriptome was used in a second pass with Cufflinks, which will quantify each transcript and gene (known or novel) in each sample.[17] The reference annotation used was from ENSEMBL 71 (Macaca Mulatta). This method allows accurate quantification of the expression of all transcripts, known or novel, with as well as previously identified LincRNAs.
